# Increased trend of thyroid cancer in childhood over the last 30 years in EU countries: a call for the pediatric surgeon

**DOI:** 10.1007/s00431-022-04596-4

**Published:** 2022-08-31

**Authors:** Claudio Spinelli, Marco Ghionzoli, Chiara Oreglio, Beatrice Sanna, Luigi De Napoli, Riccardo Morganti, Alessandro Antonelli, Antonino Morabito, Paolo Miccoli

**Affiliations:** 1 Division of Pediatric and Adolescent Surgery, Department of Surgical, Medical, Molecular Pathology and of the Critic Area, University of Pisa, Via Paradisa 2, 56124 Pisa, Italy; 2 Department of Pediatric Surgery, Meyer Children’s Hospital, University of Florence, Florence, Italy; 3 Division of Endocrine Surgery, Department of Surgical, Medical, Molecular Pathology and of the Critic Area, University of Pisa, Pisa, Italy; 4 Department of Clinical and Experimental Medicine, Section of Statistics, University of Pisa, Pisa, Italy; 5 Department of Clinical and Experimental Medicine, University of Pisa, Pisa, Italy

**Keywords:** Thyroid, Cancer, Children, Europe, Epidemiology, Surgery

## Abstract

Given the increasing incidence of thyroid cancer cases affecting the pediatric population in Europe, an epidemiological analysis of the available data is essential in order to update standards of care and recommend diagnostic and therapeutic strategies coherent to the new epidemiological trend. An observational study was conducted, considering the time interval 1991–2012, acquiring data collected by the International Agency for Research on Cancer (IARC) through the “Cancer Incidence in 5 continents plus” (CI5 plus) project and dividing patients into four groups of age (0–4, 5–9, 10–14, 15–19 years old). Data retrieved were compared to the latest report from Global Cancer Observatory, collected through the GLOBOCAN 2020 project, dividing patients in two groups: age 10–14, 15–19. Our findings highlighted an increasing prevalence of thyroid cancer rates in patients aged from 10 to 15 years old and from 15 to 19 years old, mainly in the female population, in Belarus (from 1991 to 2007), Cyprus, and Italy (from 2008 to 2012). Moreover, a further increase in incidence was highlighted in Italy according to data from GLOBOCAN 2020, with respect to other European countries.

*Conclusion*: An increasing prevalence of thyroid cancer is seen in age groups 10–19. Further research is required to understand the underlying cause and concomitantly identify the most proper screening and diagnostic techniques of thyroid nodules. Indeed, this is crucial to reach optimal surgical standards and better prognosis, possibly with more conservative approaches and maybe less postoperative complications.

**Table:** 

**What is Known:** *• Thyroid cancer is the 4th most common malignancy in children.*	
**What is New:** *•* *In the last decades, an increasing pattern has been recorded.* • *In the US, it has outlined breast cancers as the most common cancer in females.*

**Supplementary Information:**

The online version contains supplementary material available at 10.1007/s00431-022-04596-4.

## Introduction

Thyroid carcinoma (TC) is the fourth most common tumor in the pediatric population and the most frequent endocrine malignancy, especially in females [1, 2]. During the last decades, an increasing pattern in terms of incidence has been observed worldwide [3]. The exact underlying causes are far to be known, but possible explanations may reside in the increased presence of known risk factors, such as obesity and exposure to ionizing radiations. The former has been proven to be correlated to as TC development due to adiponectin secretion and leptin inhibition [4]. Moreover, in recent years, numerous reports have focused on environmental factors such as pollution, diet, and lifestyle, reporting that exposure to endocrine disruptors such as polybromurates (PDBEs) and heavy metals alter thyroid function and are associated with an increased risk of thyroid cancer [5]. It must also be underlined that the frequency of autoimmune thyroiditis, which is the most common cause of primary hypothyroidism, has increased in recent times: the underlying autoimmune process and subsequent secretion of proinflammatory cytokines promote the development of an inflammatory environment which may play a role in increasing the risk of malignancy [6]. On the other hand, an increasingly enhanced use of imaging techniques such as ultrasound as a standard diagnostic tool promoted surveillance of neck nodules and may have led to overdiagnosis of thyroid nodules in the pediatric population [7, 8].

This increasing pattern in TC has also been recorded in the USA, where in terms of proportions TC has outlined breast cancer as the most common cancer in females [1]. Increasing incidence in Europe has been observed thanks to the data collected through projects such as the Cancer Incidence 5 Plus project and the Global Cancer Observatory and International Agency for Research on Cancer, both coordinated by the World Health Organization. These projects traced the number of cases of TC worldwide and collected them on an online platform, making them accessible to everyone. In the light of the new TC trend, an epidemiological analysis of the available data regarding European countries is essential. Indeed, by analyzing the new incidence and prevalence patterns of the disease, improvements in both screening and diagnostic processes can be achieved. The latter will consequently lead to earlier diagnosis and more prompt treatment, possibly with more conservative approaches and better therapeutical strategies.

We compared data collected from 1991 to 2012 with the latest reports of the GLOBOCAN 2020 project, to evaluate the latest epidemiological trend to suggest proper screening and diagnostic strategies, eventually intended to propose better therapeutic approaches accordingly.

## Materials and methods

The incidence of TC in the following countries was considered, referring through the CI5plus database [9]: Belarus, Austria, Bulgaria, Croatia, Cyprus, Czech Republic, France (9 registers), Germany (2 registers), Italy (8 registers), Spain (9 registers). Other European countries were not taken into consideration given the absence of specific epidemiological relevance. Incidence was calculated and expressed per 100,000 inhabitants.

Data retrieved during the time frame from 1991 to 2012 divided the pediatric population into 4 groups: 0–4 years old, 5–9 years old, 10–14 years old, 15–19 years old. Data from 1991 to 1998 were available only for Belarus. With respect to Austria, Bulgaria, France, Germany, Italy, and Cyprus, no data were available for the year 1997. Italy and France are lacking data of years 2011 and 2012. Regarding TC incidence in 2020 of all the aforementioned countries instead, data were collected through the GLOBOCAN 2020 database. Patients were grouped according to age into 2 groups: 10–14 years old, 15–19 years old.

Data from males and females were analyzed separately in all cases. The data were analyzed by drawing polynomial trend lines of various degrees (from 2nd to 6th), with an *R*-squared (correlation index) > 0.5 reinforcing the correlation (not available graphics).

## Results

### Age group

#### Belarus

From 1991 to 1995, all age groups were consistently affected by TC, with female patients aged 5–9 years old presenting a peak of 8.0 cases/100,000 inhabitants in 1993. Subsequently, a rising trend was observed, involving almost exclusively the 10–14-year-old group and the 15–19-year-old group. The former reached its incidence peak in 1996 (*F*: 13.4 cases/100,000 inhabitants; *M*: 8.2 cases/100,000 inhabitants). The 15–19-year-old group instead peaked in 2001–2002, followed by a decrease until 2006 and 2007. From 2007, a steep rise until 2012 occurred.

#### Other countries

**Fig. 1:**
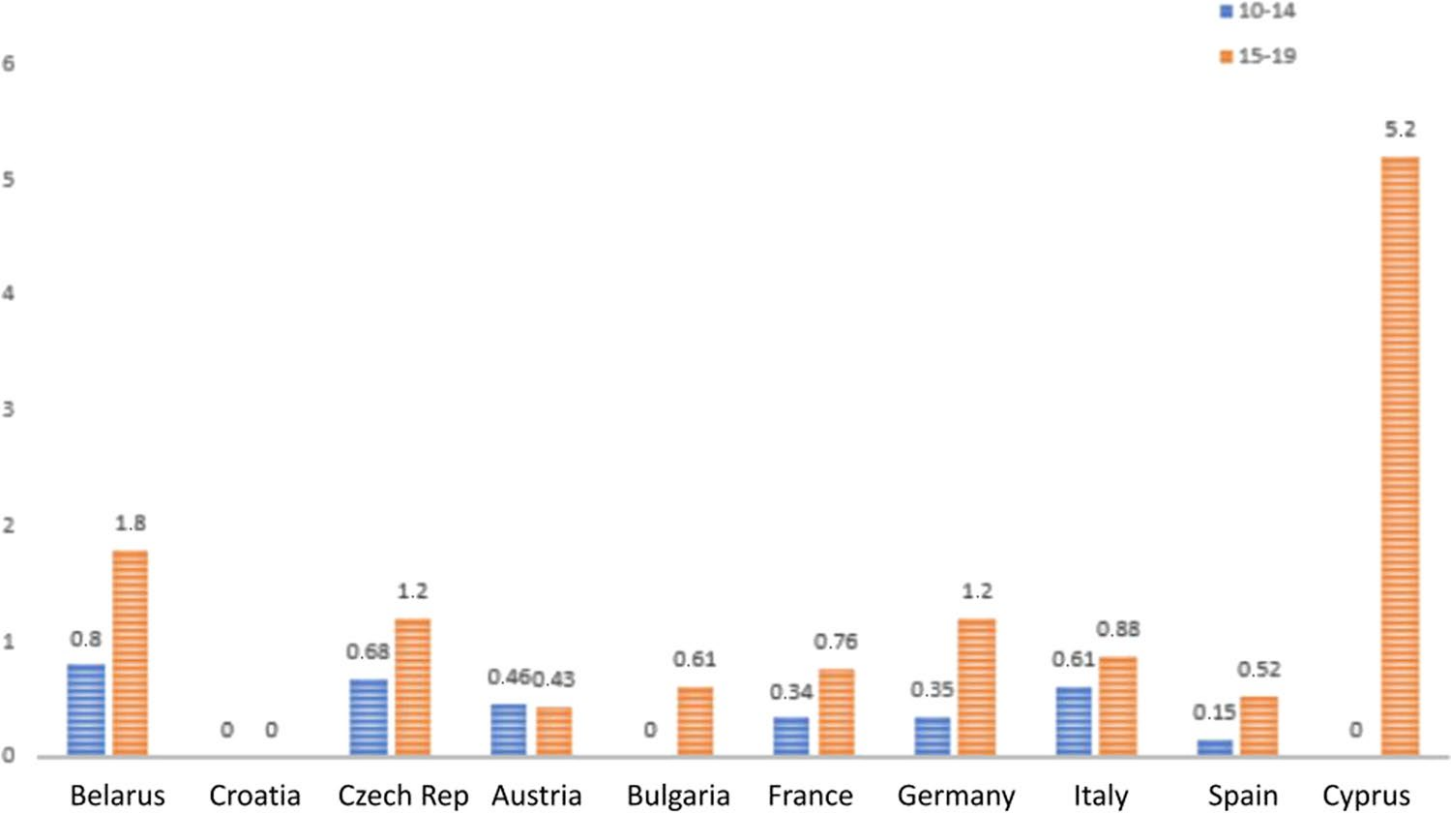
Standardized European incidence of thyroid cancer in 2020 in female population in two age groups (10–14 and 15–19 years of age). (*Data IARC—GLOBOCAN 2020*)

**Fig. 2:**
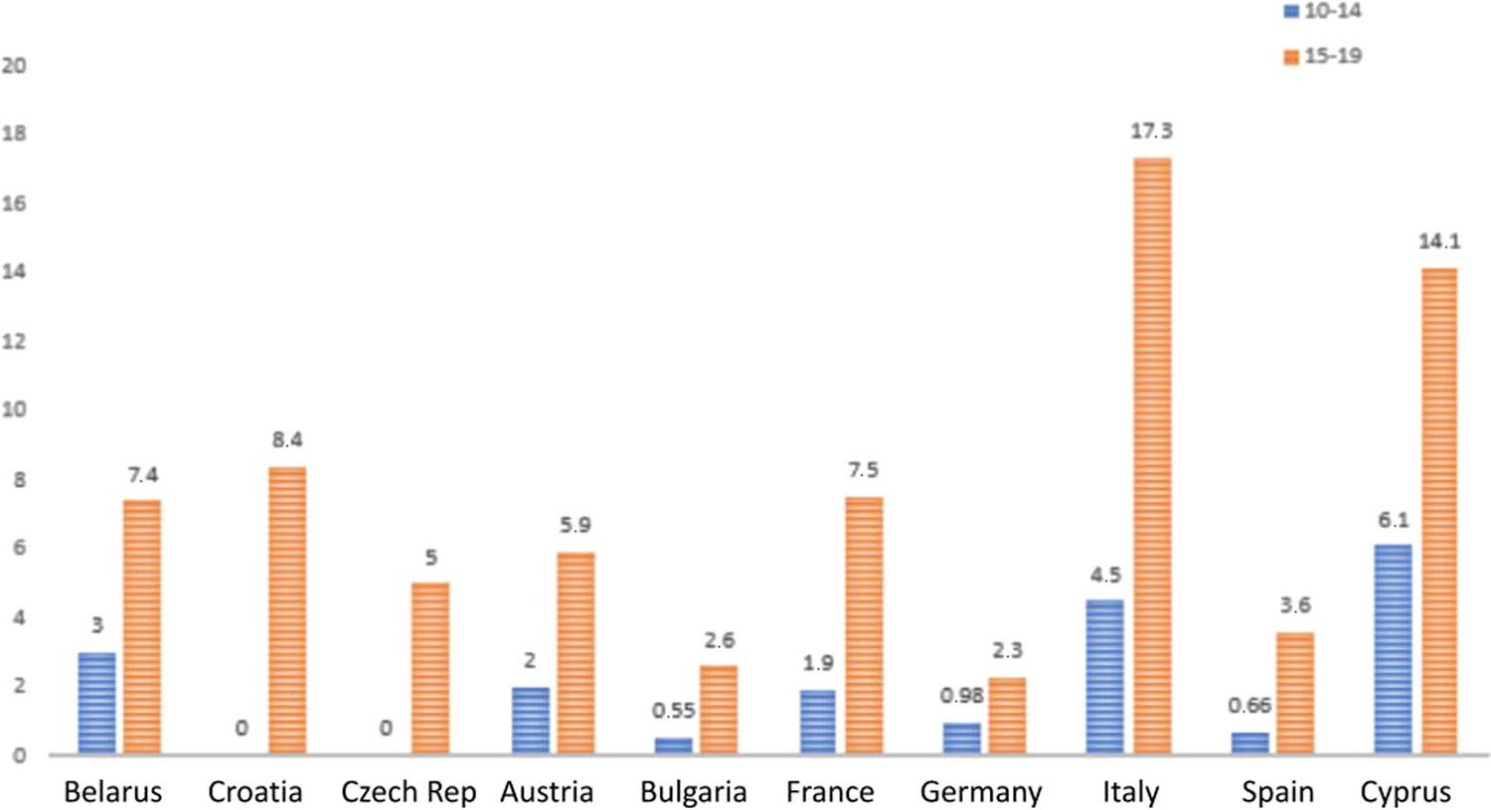
Standardized European incidence of thyroid cancer in 2020 in male population in two age groups (10–14 and 15–19 years of age). (*Data IARC—GLOBOCAN 2020*)

During the time from 1998 to 2012, the incidence of TC was almost stable in all the European countries analyzed. Data from the GLOBOCAN 2020 project highlight a substantial increase in the incidence of TC and confirm late adolescents (15–19 years old) to be the most affected population. Differences among the two age groups were subtle, with a slight increase of cases in adolescents aged 15–19 years old. In 2020, major differences were found with respect to the age group considered: female late adolescents indeed presented a much higher incidence with respect to girls aged 10–14 years old. Regarding the latter group, Croatia and Czech Republic recorded 0 new cases in 2020 (Figs. 1 and 2).

### Gender differences

#### Belarus

From 1991 to 2012, the incidence of TC in Belarus presented a similar trend in both the female and male populations. The pattern of distribution was almost superimposable for both genders in all age groups. In the age group 10–14 years old, the incidence in both genders decreased from 1996 to 2001. Then, a steady increase began and continued until 2012.

Instead, the population aged 15–19 years old presented a marked difference between the two genders: in 2001–2002, the incidence in male was 6.2 cases/100,000 inhabitants), while females almost doubled it with 14.8 cases/100,000 inhabitants (Supplementary Fig. 1).

#### Other countries

**Fig. 3:**
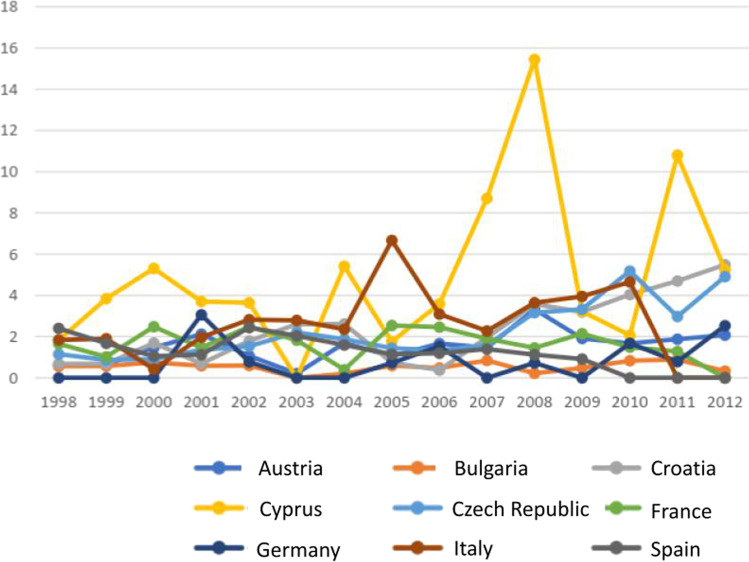
Thyroid carcinoma incidence in EU countries, female population, age group 10–19. (*Data collected from IARC 1998–2012)*

**Fig. 4:**
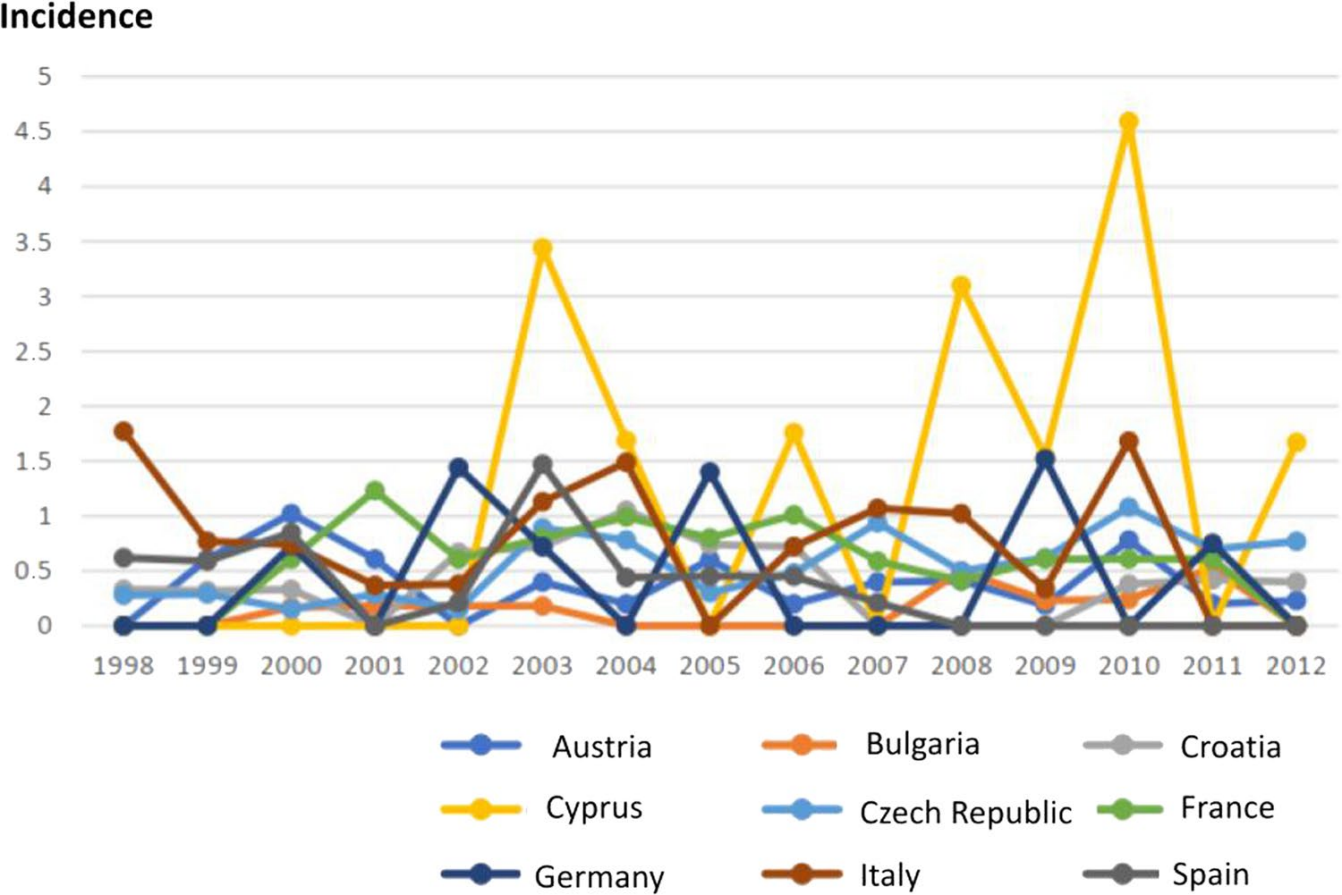
Thyroid carcinoma incidence in EU countries, male population, age group 10–19. (*Data collected from IARC 1998–2012)*

From 1998 to 2012, a slightly increasing trend was observed in Croatia, Czech Republic, Italy, and Germany with respect to the female population, while the incidence decreased in France and Bulgaria. Regarding the male population instead, Czech Republic and Bulgaria recorded an increased number of cases, while Spain, Italy, and France registered a decreasing pattern (Figs. 3 and 4). Regarding the male population, an incidence peak occurred in Cyprus (5.2 cases/100,000 inhabitants), while the other countries presented a comparable incidence.

In 2020, in the female population, the highest incidence was recorded in Italy (17.3 cases/100,000 inhabitants), followed by Cyprus (14.1 cases/100,000 inhabitants) and Croatia (8.4 cases/100,000 inhabitants). Findings regarding this group were consistent also regarding other countries, with Belarus presenting > 7.4 cases/100,000 inhabitants and France having 7.5 cases/100,000 inhabitants.

The global incidence of thyroid cancer in European countries in 2020 presents some interesting data, such as an increase of almost tenfold in the female population in Italy (10.1 cases/100,000 inhabitants), in Croatia (6.1 cases/100,000 inhabitants), and in France (4.1 cases/100,000 inhabitants). Regarding males instead, Cyprus reached 2.6 cases/100,000 inhabitants and Czech Republic estimated almost 1 case/100,000 (Supplementary Figs. 2 and 3).

## Discussion

The incidence of thyroid cancer has progressively increased worldwide [10]. This analysis presents Belarus, Cyprus, and Italy as the countries mostly affected by thyroid cancer in the pediatric population. In addition, it must be emphasized that Italy presents a greater number of cases when compared to other countries, with the exception of Cyprus. Conversely, Belarus shows a stable high incidence during all time periods evaluated in the present study. The causes behind this increasing pattern are still a matter of debate in the scientific community and they are most likely multifactorial. The exposure to ionizing radiation is a well-established and documented risk factor for thyroid cancer development, as demonstrated by the surge of thyroid cancer following the Chernobyl accident in 1986 [11, 12]. Iodine deficiency causes thyroid stimulation upon TSH secretion which ultimately promotes follicular cell growth [13]. Our study supports this theory from an epidemiological point of view. Belarus data showing an increase of thyroid cancer cases in all age groups, even patients aged 0–4 years old who are hardly ever involved in the other countries, was observed, likely due to the increased population’s exposure to ionizing radiations [12, 14]. The correlation is striking when looking at the pattern of distribution of thyroid cancer, given the high number of cases recorded in the years following the accident.

Furthermore, also a complex mechanism involving estrogen signaling has been demonstrated to play a role in thyrocyte proliferation: hormones have an indirect effect through increased secretion of thyroxine binding globulin and a direct effect through binding to estrogen receptors (ERα and ERβ) present in normal and neoplastic thyroid cells [15, 16]. As for Cyprus and Italy, data show a significant number of cases and females seem to be more affected in the 10–14 and 15–19 age groups, while males appear to be mostly involved when aged 15–19 years old. A sensible difference between the two genders emerges, supporting the plausible role of estrogens in the development of thyroid cancer [17]. This hypothesis is further supported by the much higher incidence of the condition in females aged 15–19 years old compared to females aged 10–14 years old. However, Lebbink et al. [18] found a female predominance in differentiated TC even in prepubertal girls < 10 years old, who had not been exposed to a surge of estrogen levels yet. This finding is controversial, since the vast majority of recent works claimed that increased age results in an increasingly disproportionate number of females affected compared to their male peers, with nearly a 6:1 ratio by 15–19 years of age [19]. The underlying cause is speculated to be due to estrogen exposure during mini-puberty or estrogen derived from adipose tissue, as suggested by Derwahl and Nicula, Paulson et al., and Tawde and Jeyakumar [17, 19, 20].

An interesting aspect to be considered is that differently from adults, thyroid nodules are more rarely palpable in children, leading to delayed diagnosis at a more advanced stage of disease. Moreover, pediatric thyroid nodules carry a 2-to-fivefold higher risk of malignancy [21] and present differences in terms of histological and oncogenic profiles with respect to adult series [22–26]. Also, treatment-related morbidity remains a substantial element [27]. Indeed, it is well known that thyroidectomy can be associated with a considerable rate of complications such as hypoparathyroidism and recurrent laryngeal nerve injury. For all of these reasons, a more conservative approach such as hemithyroidectomy should be preferred when feasible, to reduce the aforementioned risks [28–31], especially if dealing with early-stage diagnosis and small nodules. Thus, early diagnosis is crucial to be performed and, to do so, a high suspicion level must be maintained given the most recent epidemiological trends.

It must be noted that this study presents some limitations, such as the lacking data of other European countries rather than Belarus prior to 1998. Therefore, a comparison between the peak occurred in that period of time cannot be properly performed, arising some sort of bias in the interpretation of the available data. Moreover, in the database analyzed, no data were available regarding the histopathological characteristics of TC; therefore, deeper studies should be conducted.

## Conclusions

Data interpolation demonstrated an increasing trend in the incidence of thyroid cancer in the adolescent population during the last 30 years. This data is particularly relevant in the most recent years in Italy.

Further correlational studies will be required aiming at understanding the underlying causes for this phenomenon, such as increased ultrasound screening or environmental factors. A timely diagnosis of thyroid carcinoma allows a prompt surgical treatment with a more conservative approach, less postoperative complications, and better prognosis. To sum up, the data analysis underlines the necessity to develop specific management strategies for the pediatric population, in order to face the increasing rates of thyroid cancers in the pediatric field and to offer optimal surgical treatment and avoid unnecessary invasive approaches.

## Supplementary Information

Below is the link to the electronic supplementary material.

Supplementary file1 (JPG 528 kb)

Supplementary file2 (JPG 691 kb)

Supplementary file3 (JPG 709 kb)
