# Supplementary figures and images for: Increased trend of thyroid cancer in childhood over the last 30 years in EU countries: a call for the pediatric surgeon
Source: Eur J Pediatr. 2022 Aug 31;181(11):3907–13. doi: 10.1007/s00431-022-04596-4 (PMC9546957; doi:10.1007/s00431-022-04596-4)

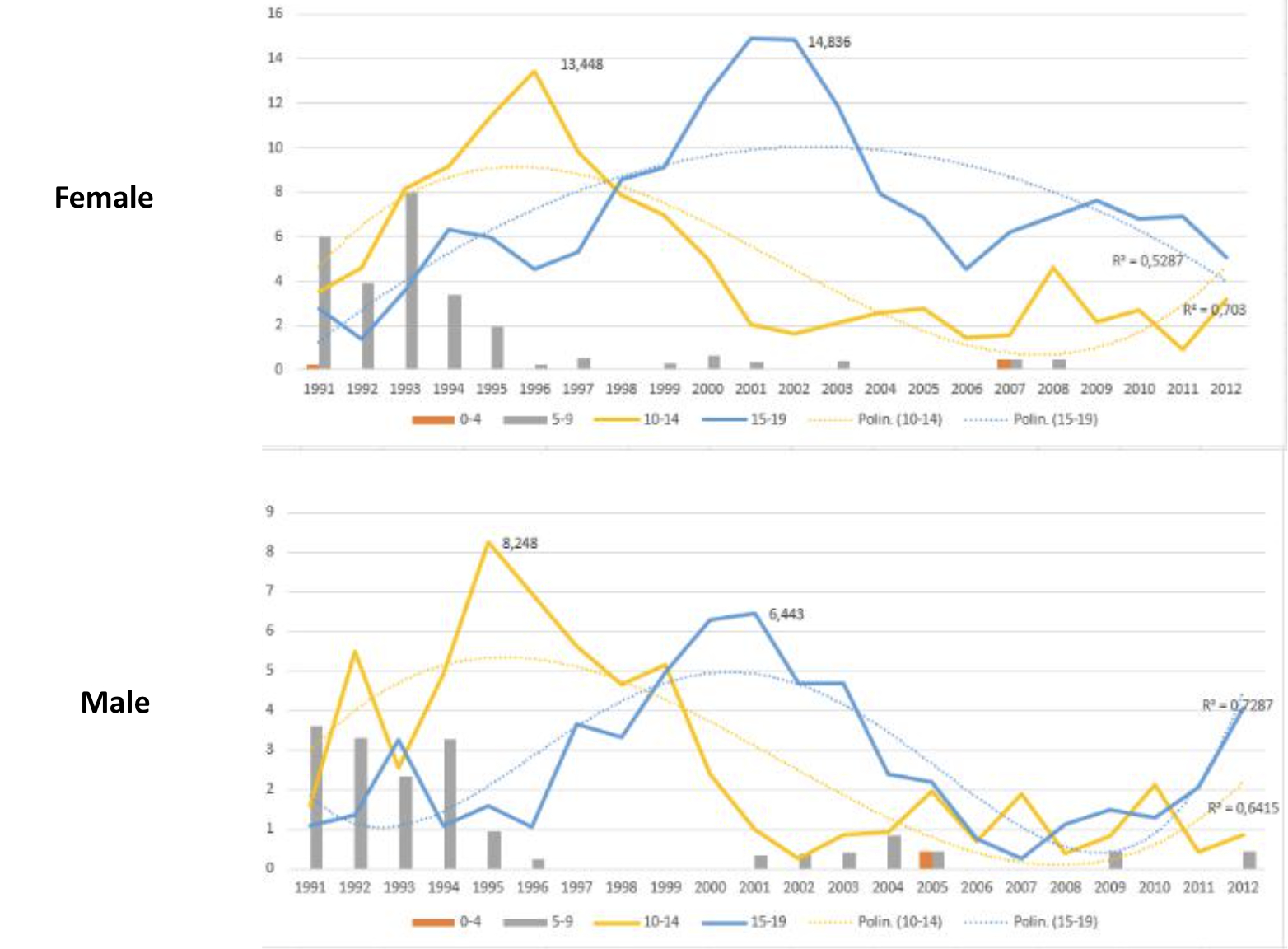

Supplement: Supplementary file 1 — Supplementary file1 (JPG 528 kb) [file 431_2022_4596_MOESM1_ESM.jpg]

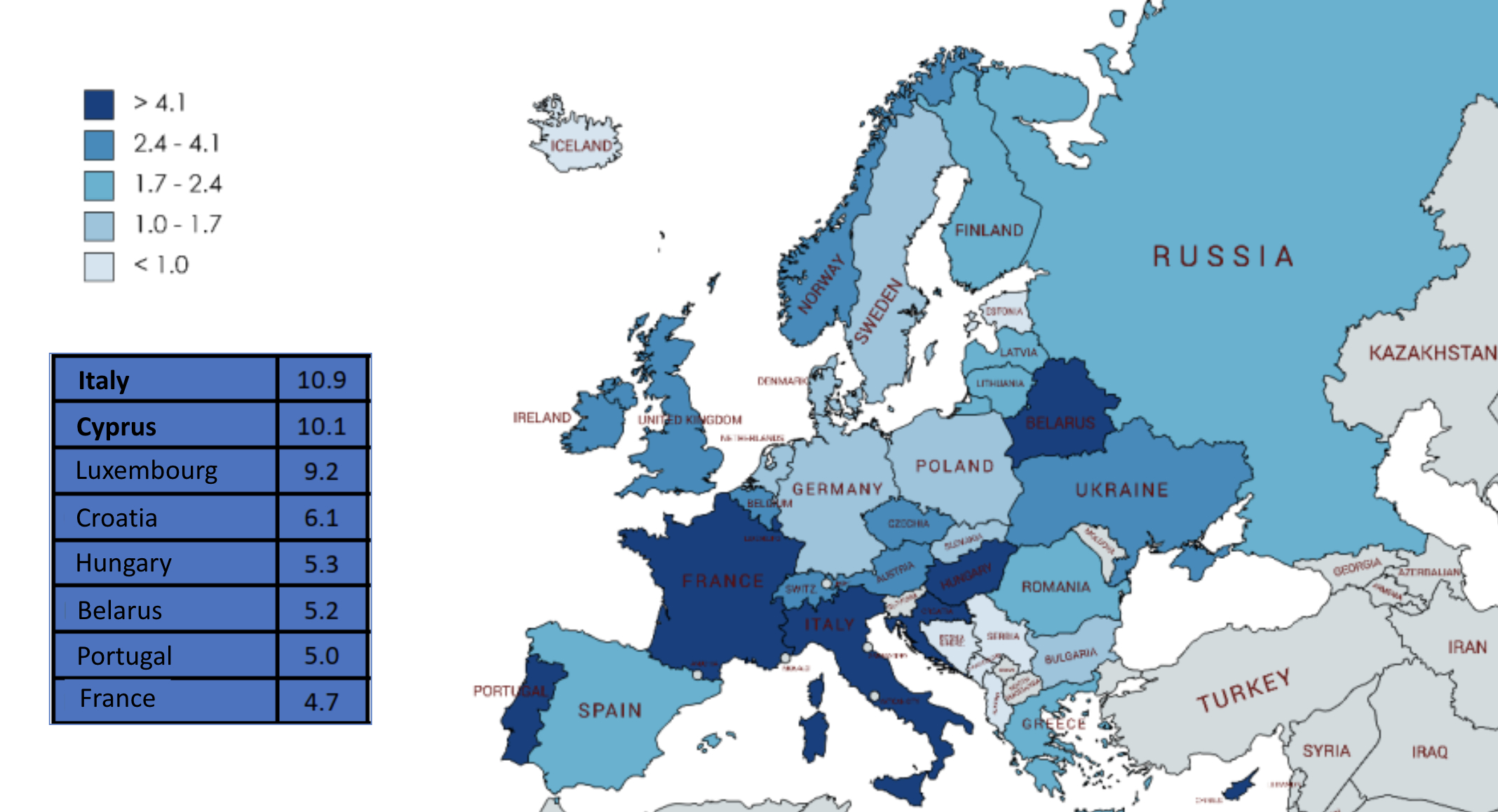

Supplement: Supplementary file 2 — Supplementary file2 (JPG 691 kb) [file 431_2022_4596_MOESM2_ESM.jpg]

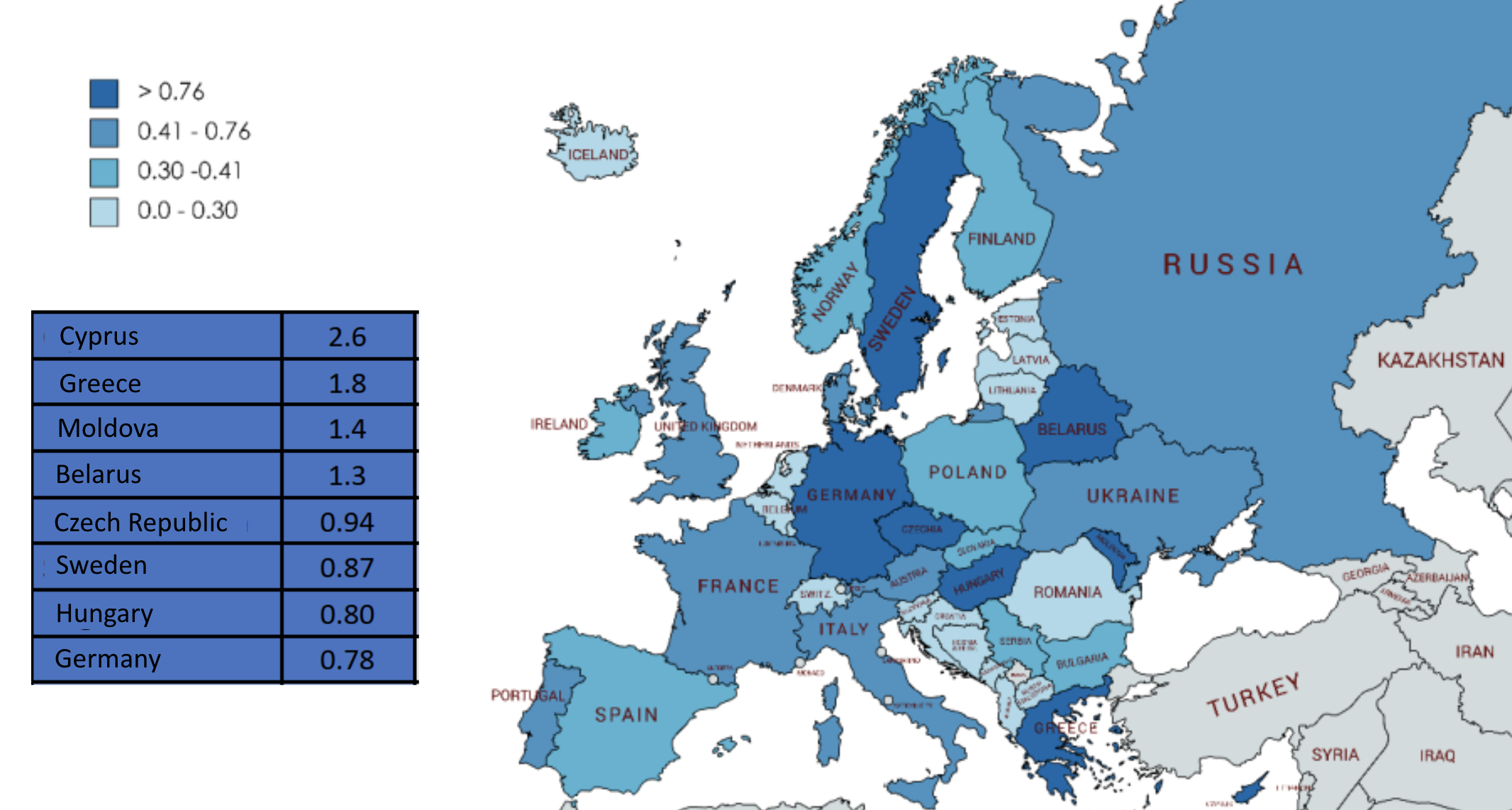

Supplement: Supplementary file 3 — Supplementary file3 (JPG 709 kb) [file 431_2022_4596_MOESM3_ESM.jpg]
